# Prevalence of neurotoxicity symptoms among postpartum women on isoniazid preventive therapy and efavirenz-based treatment for HIV: an exploratory objective of the IMPAACT P1078 randomized trial

**DOI:** 10.1186/s12884-022-05341-3

**Published:** 2023-01-17

**Authors:** Patricia Mandima, Kristin Baltrusaitis, Grace Montepiedra, Lisa Aaron, Jyoti Mathad, Carolyne Onyango-Makumbi, Mandisa Nyati, James Ngocho, Gift Chareka, Ponego Ponatshego, Gaerolwe Masheto, Katie McCarthy, Patrick Jean-Philippe, Amita Gupta, Lynda Stranix-Chibanda, for the IMPAACT P1078 T. B. APPRISE study team, Haroon Saloojee, Wafaa El-Sadr, David Harrington, Jonathan B. Levine, Mary Faith Marshall, Lucky Mokgatlhe, Paula Munderi, Andrew Nunn, Jerome Amir Singh, Betty Kwagala, Alwyn Mwinga, Papa Salif Sow, Catherine Hill, Jerrold J. Ellner, Grace John-Stewart, Steven Joffe, Barbara E. Murray, Merlin L. Robb, Enid Kabugho, Deo Wabwire, Hellen Kaganzi, Joel Maena, Hajira Kataike, Emmie Marote, Mercy Mutambanengwe, Teacler Nematadzira, Suzen Maonera, Vongai Chanaiwa, Tapiwa Mbengeranwa, Sukunena Maturure, Tsungai Mhembere, Nasreen Abrahams, Haseena Cassim, Ruth Mathiba, Joan Coetzee, Jeanne Louw, Marlize Smuts, Lindie Rossouw, Magdel Rossouw, Celeste de Vaal, Sharon Mbaba, Karen du Preez, Frieda Verheye-Dua, Aisa Shao, Boniface Njau, Philoteus Sakasaka, Seleman Semvua, Tebogo J. Kakhu, Thuto Ralegoreng, Ayotunde Omoz-Oarhe, Unoda Chakalisa, Nishi Suryavanshi, Sandesh Patil, Neetal Nevrekar, Renu Bharadwaj, Vandana Kulkarni, Fuanglada Tongprasert, Tavitiya Sudjaritruk, Chintana Khamrong, Prapaporn Janjing, Marie Flore Pierre, Maria Linda Aristhomene, Dominique Lespinasse, Emelyne Dumont, Rebecca LeBlanc, Amy James Loftis, Soyeon Kim, David Shapiro, Camlin Tierney, Vivian Rexroad, Renee Browning

**Affiliations:** 1 University of Zimbabwe Clinical Trials Research Centre, Harare, Zimbabwe; 2 Center for Biostatistics in AIDS Research, Harvard T.H. Chan School of Public Health, Boston, MA USA; 3 Weill Cornell Medical College, New York, NY USA; 4 Makerere University-John Hopkins University Research Collaboration, Kampala, Uganda; 5 Chris Hani Baragwanath Hospital, Johannesburg, Soweto, South Africa; 6 Kilimanjaro Christian Medical University College, Moshi, Tanzania; 7 Harvard AIDS Institute, Gaborone, Botswana; 8 FHI 360, Durham, NC USA; 9 National Institute of Allergy and Infectious Diseases, Bethesda, MD USA; 10 Division of Infectious Diseases, Johns Hopkins University School of Medicine, Baltimore, USA; 11 Child and Adolescent Health Unit, Faculty of Medicine and Health Sciences, University of Zimbabwe, Harare, Zimbabwe

**Keywords:** Depression, Isoniazid preventive therapy, Efavirenz, Pregnant and Breastfeeding Women, PHQ-9

## Abstract

**Background:**

This exploratory analysis investigates the prevalence and risk factors of neurocognitive toxicity in postpartum women on HIV treatment in response to a concern of an Isoniazid Preventive Therapy (IPT)/Efavirenz interaction.

**Trial Design:**

Pregnant women on HIV treatment from countries with high TB prevalence were randomized in IMPAACT P1078 to 28 weeks of IPT started either during pregnancy or at 12 weeks postpartum. Partway through study implementation, the Patient Health Questionnaire 9, the cognitive complaint questionnaire, and the Pittsburg Sleep Quality Index were added to evaluate depression, cognitive function, and sleep quality at postpartum weeks. Screening for peripheral neuropathy was conducted throughout the study.

**Methods:**

We summarized percentages of women with depression symptoms, cognitive dysfunction, poor sleep quality and peripheral neuropathy and assessed the association of 11 baseline risk factors of neurotoxicity using logistic regression, adjusted for gestational age stratum.

**Results:**

Of 956 women enrolled, 749 (78%) had at least one neurocognitive evaluation. During the postpartum period, the percentage of women reporting at least mild depression symptoms, cognitive complaint and poor sleep quality peaked at 13%, 8% and 10%, respectively, at 12 weeks, and the percentage of women reporting peripheral neuropathy peaked at 13% at 24 weeks. There was no evidence of study arm differences in odds of all four neurotoxic symptoms.

**Conclusions:**

Timing of IPT initiation and EFV use were not associated with symptoms of neurotoxicity. Further study is advised to formally assess risk factors of neurotoxicity.

**Supplementary Information:**

The online version contains supplementary material available at 10.1186/s12884-022-05341-3.

## Background

Isoniazid (INH) Prevention Therapy (IPT) is recommended by the World Health Organization (WHO) for pregnant women living with HIV in countries with high tuberculosis (TB) prevalence [1], many of whom also receive Efavirenz-based antiretroviral treatment (EFV-ART). Both INH and EFV are independently associated with neurotoxicity. INH-related peripheral neuropathy resulting from its effect on pyridoxine-dependent coenzymes is well described [2], while INH-related neurocognitive toxicity effects include memory loss, depression, sleep disturbances and acute psychosis [3]. EFV has high cerebral-penetration effectiveness [4], readily crossing the blood–brain barrier and being concentrated in brain tissue [5]. Central nervous system effects appear soon after initiation of EFV, may be transient, and include abnormal dreams, sleep disturbances, anxiety and depression [3].

As both EFV and INH are metabolized in the liver, drug-drug interactions have been noted [4, 6]. Specifically, INH inhibits efavirenz 7-hydroxylation by the CYP2A6 enzyme through mechanism-based inactivation resulting in higher plasma EFV concentrations with concomitant use [6]. However, this drug-drug interaction is genotype dependent. Studies have shown that in pregnant women, INH use decreased EFV clearance by 7% in normal EFV metabolizers and 13% in slow/intermediate EFV metabolizers [7]. Although the frequency of side effects was not assessed, sustained, high EFV concentrations could lead to an increased susceptibility to drug-related toxicity [3]. This potential increase is alarming because neuropsychiatric conditions in postpartum women are associated with undesirable effects on maternal-infant relationship development and child development [8, 9].

Despite these known risks, the frequency of neurocognitive toxicity due to INH, EFV or both is less established in postpartum women, a time already characterized by sleep disturbance and increased risk for depression [8]. Although Dolutegravir (DTG)-containing ART is now the recommended first line regimen [10], many women are yet to transition to DTG-ART and those who do not tolerate DTG may take EFV-based ART. Therefore, understanding the prevalence and risk factors of neurocognitive symptoms experienced by postpartum women living with HIV taking EFV or EFV/INH is important. In response to these concerns, an exploratory objective was added to the multi-country IMPAACT P1078 clinical trial to determine the proportion of pregnant or breastfeeding women on ART (EFV-ART, NVP-ART or other) and randomized to initiate IPT in pregnancy or postpartum, who reported symptoms of neurocognitive toxicity (depression symptoms, cognitive complaint and poor sleep quality) or peripheral neuropathy. Associations of socio-demographic and medical factors with neurocognitive toxicity as well as peripheral neuropathy were also evaluated, with particular interest in EFV exposure and its potential for treatment effect modification.

## Methods

### Study design

IMPAACT P1078 TB APPRISE [ClinicalTrials.gov number, NCT01494038, first registered on 16/12/2011, (https://clinicaltrials.gov/ct2/show/NCT01494038)] was a Phase IV, randomized, double-blind, placebo-controlled non-inferiority study that compared the overall safety and risks and benefits of immediate versus deferred IPT in pregnant women living with HIV, on ART and their infants, enrolled at ≥ 14 through ≤ 34 weeks gestation, at high risk for TB. It was a multi-site study performed in Thailand, Haiti, India, Uganda, Tanzania, South Africa, Botswana and Zimbabwe. At study entry, women were randomized in a 1:1 ratio to initiate oral IPT either during pregnancy (Immediate INH) or at week 12 after delivery (Deferred INH). Women in the Immediate arm were given 300 mg of INH daily initiated at study entry and continued for 28 weeks followed by a Placebo for INH until 40 weeks postpartum. Women in the Deferred arm were given a Placebo for INH initiated at study entry followed by 300 mg of INH daily initiated at 12 weeks postpartum and continued for 28 weeks. All women were provided with pyridoxine (vitamin B_6_) and prenatal multivitamins from study entry until 40 weeks postpartum. Non-study standard-of-care Cotrimoxazole prophylaxis was provided to women with CD4 counts below 350 cells/mm [3]. Women and their infants were followed through 48 weeks postpartum. Randomization was stratified by site and gestational age (≥ 14 weeks to < 24 weeks or ≥ 24 weeks to ≤ 34 weeks). Full details of the P1078 study design have been described elsewhere [11] and can be found here: https://www.impaactnetwork.org/studies/p1078.

The analysis of neurocognitive toxicity of INH and EFV was informed by questions from three assessments added to protocol Version 2.0. The Patient Health Questionnaire 9 (PHQ-9) [12] captured depression symptoms over the previous two weeks. A three-item questionnaire adapted from Simioni et al. [13] assessed cognitive complaints. Finally, several questions were adapted from the Pittsburgh Sleep Quality Index (PSQI) to assess sleep habits during the past month [14]. Because the neurocognitive toxicity evaluations were not included until partway through study implementation, these evaluations were not available for every woman at every study visit. However, peripheral neuropathy, which was evaluated using the Brief Peripheral Neuropathy Screening (BPNS) tool [15], was assessed throughout the duration of the study.

### Participants

The neurocognitive analysis set included all women who were randomized and had at least one neurocognitive evaluation during follow-up. Analyses that assess peripheral neuropathy included all randomized women.

### Trial procedures

Neurocognitive toxicity (including depression symptoms, cognitive complaint and poor sleep quality) was assessed at entry, every 12 weeks during the antepartum (AP) period and postpartum (PP) weeks 4, 12, 24, 36 and 48. Peripheral neuropathy was assessed at entry, every 4 weeks during the AP period, labor and delivery and every 4 weeks during the PP period. Analysis visits were defined and labeled as the antepartum visit closest to delivery (AP visit), postpartum study visits at weeks 4, 12, 24, 36 and 48 (PP visits).

### Outcome measures

#### Depression symptoms

Depression was measured using the PHQ-9 assessment, which is a reliable depression screening tool validated in many languages including those used in the P1078 study [12, 16]. Probable depression was defined as total PHQ-9 score ≥ 10. Depression symptoms were also evaluated using an ordinal scale, classifying the total PHQ-9 score as no depression (0), minimal depression (1–4), mild depression (5–9), moderate depression (10–14) and severe depression (≥ 15) [17, 18].

#### Cognitive complaint

Participants were classified as having a cognitive complaint if they responded “Yes, Definitely” to any of three questions relating to the neurocognitive impairment questions:

1. Do you experience frequent memory loss?
2. Do you feel that you are slower when reasoning, planning activities or solving problems?
3. Do you have difficulties paying attention?

Incomplete answers without at least one “Yes, Definitely” response were set to missing.

#### Poor sleep quality

Poor sleep quality was assessed using an adapted PSQI (aPSQI) assessment. The PSQI is a reliable and valid tool for measuring sleep quality and disturbance in multiple populations [15]. A total aPSQI score ≥ 4 indicated “poor” sleep quality. If one response was missing, then the total score was set to missing [19].

#### Peripheral neuropathy

Peripheral neuropathy was assessed using the BPNS tool [15, 16]. This screening tool evaluated symptoms in the feet and legs, vibration perception through the interphalangeal bone of the great toe and deep tendon reflexes in the Achilles tendon. The Grade of peripheral neuropathy at each visit was based on the final BPNS score, where Grade 0 (normal or currently absent) was defined as final BPNS score = 0, Grade 1 (mild) was 1–3, Grade 2 (moderate) was 4–6 and Grade 3 (severe) was ≥ 7 [20]. Peripheral neuropathy was defined as Grade ≥ 1.

Additional details for all outcome measures are provided in Supplementary Fig. 1.

### Statistical analysis

Analyses were carried out using an intent to treat approach (analyzed as randomized). The two randomization arms are labeled as Immediate INH and Deferred INH. Unless otherwise noted, baseline values refer to the last determination before or on the randomization date. Baseline characteristics were not compared across arms.

### Prevalence of neurotoxicity

Frequencies and percentages of women with depression symptoms, cognitive complaint, poor sleep quality and peripheral neuropathy were summarized at each analysis visit, with Clopper-Pearson 95% confidence intervals (CI) for dichotomous outcome measures.

### Study arm differences in neurotoxicity

Logistic regression, adjusted for gestational age stratum, was used to compare the Immediate and Deferred INH arms with respect to cognitive complaint, poor sleep quality and peripheral neuropathy at each analysis visit. Given the small number of probable depression events, exact logistic regression was used for this outcome measure. Because potential interaction effects between timing of INH initiation and EFV exposure were of particular interest, a supplemental analysis summarized neurotoxicity by EFV exposure.

### Factors associated with neurotoxicity

Exact logistic regression models were used to assess risk factors of probable depression, cognitive complaint and poor sleep quality at PP Week 36. This time point was selected ad hoc to maximize power (the largest number of women reported probable depression at this time point). Logistic regression models were also used to assess risk factors of peripheral neuropathy at any visit. First, each risk factor was included in a model adjusted only for gestational age stratum. Then, covariates from the simple models with *P* values < 0.15 and gestational age stratum were entered into a multivariable adjusted analysis, unless modeling became unstable. Risk factors were assessed at entry and included study arm, EFV-regimen, hepatitis B surface antigen (HBsAg) status, hepatitis C serology status, country, CD4 count, HIV viral load, age, body mass index (BMI), INH acetylation status, EFV metabolism status and Cotrimoxazole use. Estimates of covariate effects in multivariate models with 95% CIs are presented.

### Incidence of neurotoxicity

Incidence and 95% CIs in neurocognitive toxicity (probable depression, cognitive complaint and poor sleep quality) were calculated by study arm and overall for the subset of women who had at least one neurocognitive toxicity evaluation at both an antepartum and a postpartum visit. For these analyses, the neurocognitive toxicity evaluation at the antepartum visit closest to delivery was used as baseline, and women were censored at their last postpartum neurocognitive toxicity evaluation. Incidence and 95% CIs of peripheral neuropathy were calculated based on data from all women, and the peripheral neuropathy evaluation closest to randomization was used as baseline.

Because these analyses were exploratory, there were no adjustments for multiple testing. Results with *P* values < 0.05 were considered significant. All analyses were performed using SAS 9.4.

## Results

### Accrual and analysis exclusions

**Fig. 1:**
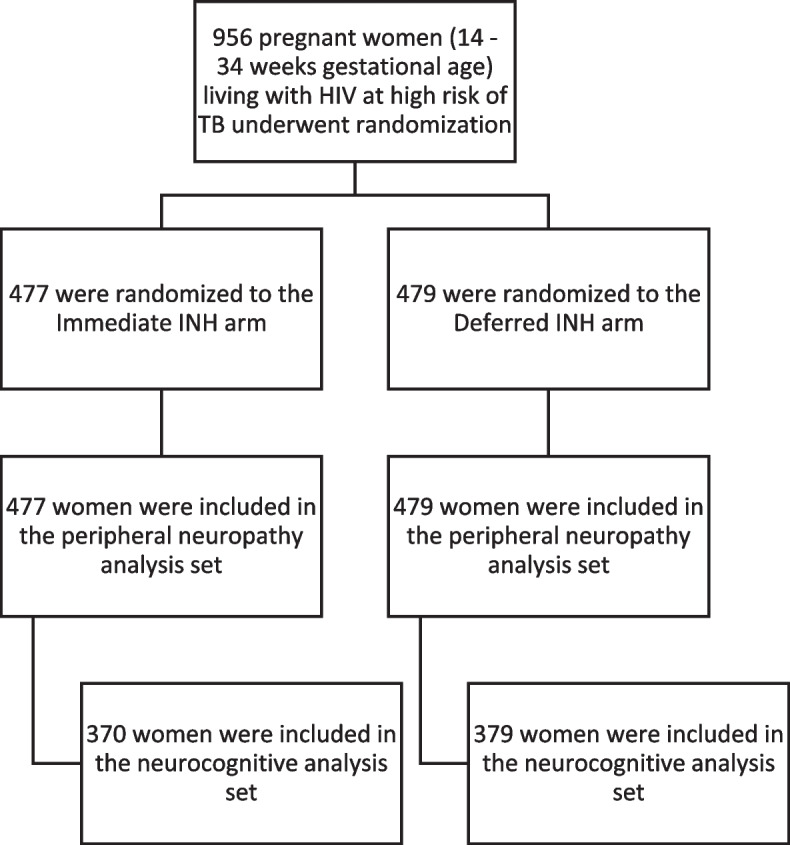
Randomization and analysis sets

A total of 956 women were enrolled in P1078 between August 2014 and April 2016 at 13 sites in eight countries. Of the women enrolled in P1078, 749 women, 370 in the Immediate INH arm and 379 in the Deferred INH arm, had at least one neurocognitive toxicity evaluation. These women were included in the neurocognitive analysis set (Fig. 1).

### Baseline characteristics

For the neurocognitive analysis set, baseline demographic and health characteristics were similar across the two randomization arms (Table 1). Of note, baseline neurocognitive assessments were not available for all women, and baseline peripheral neuropathy with severity of at least Grade 1, per the Division of AIDS (DAIDS) toxicity table, was an exclusion-criteria for the study. Baseline demographic and health characteristics were also similar between the peripheral neuropathy (i.e., all enrolled participants) and the neurocognitive analysis sets (Supplemental Table 1).

**Table 1:** Maternal characteristics at baseline for the neurocognitive analysis population. Continuous variables are presented as median (25^th^, 75^th^ percentile), and categorical variables are presented as N (%) or N/Total N (%) for variables with missing values

		**Immediate INH** **(*N*** **= 370)**	**Deferred INH** **(*N*** **= 379)**	**Overall** **(*N*** **= 749)**
Age (years)	Median (25^th^, 75^th^)	29.0 (25.0, 33.0)	29.0 (24.0, 34.0)	29.0 (24.0, 33.0)
	16—< 20	14 (4)	17 (4)	31 (4)
	20—< 24	61 (16)	69 (18)	130 (17)
	≥ 24	295 (80)	293 (77)	588 (79)
Self-reported race/ethnicity	Black African	337 (91)	335 (88)	672 (90)
	Black of African origin	7 (2)	12 (3)	19 (3)
	Indian or South Asian	13 (4)	13 (3)	26 (3)
	Thai	8 (2)	12 (3)	20 (3)
	Other Asian	5 (1)	7 (2)	12 (2)
Country	Botswana	29 (8)	30 (8)	59 (8)
	Haiti	7 (2)	12 (3)	19 (3)
	India	13 (4)	13 (3)	26 (3)
	South Africa	65 (18)	63 (17)	128 (17)
	Tanzania	32 (9)	33 (9)	65 (9)
	Thailand	13 (4)	18 (5)	31 (4)
	Uganda	68 (18)	64 (17)	132 (18)
	Zimbabwe	143 (39)	146 (39)	289 (39)
Gestational age (weeks)	14—< 24	130 (35)	136 (36)	266 (36)
	24—34	240 (65)	243 (64)	483 (64)
HIV-1 RNA (copies/mL)	Undetectable	226 (61)	241 (64)	467 (62)
CD4 count (cells/mm^3^)	Median (25^th^, 75^th^)	495 (348, 679)	505 (367, 703)	499 (355, 689)
BMI (kg/m^2^)	Median (25^th^, 75^th^)	26.3 (23.4, 30.3)	26.1 (23.1, 29.5)	26.2 (23.3, 29.9)
WHO clinical staging for HIV	Clinical stage I	327 (88)	331 (87)	658 (88)
IGRA positivity	Positive	104/363 (29)	114/373 (31)	218/736 (30)
	Negative	235/363 (65)	233/373 (62)	468/736 (64)
	Indeterminate	24/363 (7)	26/373 (7)	50/736 (7)
HBsAg serology	Positive	12 (3)	14 (4)	26 (3)
Hepatitis C serology	Positive	5/345 (1)	4/353 (1)	9/698 (1)
INH acetylation status	Fast	59/361 (16)	58/348 (17)	117/709 (17)
	Intermediate	139/361 (39)	140/348 (40)	279/709 (39)
	Slow	163/361 (45)	150/348 (43)	313/709 (44)
EFV metabolism status	Fast	89/358 (25)	91/363 (25)	180/721 (25)
	Intermediate	191/358 (53)	194/363 (53)	385/721 (53)
	Slow	78/358 (22)	78/363 (21)	156/721 (22)
Cotrimoxazole use	Yes	179/369 (48)	164/378 (43)	343/747 (46)
ARV regimen	EFV	313 (85)	327 (86)	640 (85)
	NVP	46 (12)	46 (12)	92 (12)
	Other	11 (3)	6 (2)	17 (2)
Duration of EFV-containing regimen prior to entry (weeks)^a^	Median (25^th^, 75^th^)	10.0 (3.0, 41.0)	9.0 (3.0, 30.0)	9.0 (3.0, 38.5)

*INH* Isoniazid, *BMI* Body mass index, *WHO* World health organization, *IGRA* Interferon gamma release assay, *HBsAg* Hepatitis B surface antigen, *EFV* Efavirenz, *ARV* Antiretroviral

^a^Among women on EFV-containing regimen

### Treatment status

At the PP week 12 visit, approximately two-thirds of women in the Immediate INH arm were still taking INH (77/113) and most women in the Deferred INH arm initiated INH (100/119; Supplemental Table 2).

**Table 2:** Summary of neurotoxicity (depression symptoms, cognitive complaint, poor sleep quality, and peripheral neuropathy Grade) by postpartum study visit. Values are presented as N/Total N [% (95% CI)] for dichotomous outcome measures and N/Total N (%) otherwise

Analysis Visit	Depression Symptoms^a^				Cognitive Complaint^b^	Poor Sleep Quality^c^	Peripheral Neuropathy^d^		
	**Minimal** **(PHQ-9: 1–4)**	**Mild** **(PHQ-9: 5–9)**	**Moderate** **(PHQ-9: 10–14)**	**Moderately severe, severe** **(PHQ-9: 15–27)**	**Yes**	**Yes**	**Mild** **(Grade 1)**	**Moderate (Grade 2)**	**Severe (Grade 3)**
AP Visit	27/78 (35)	11/78 (14)	0/78 (0)	0/78 (0)	5/78 [6 (2, 14)]	11/73 [15 (8, 25)]	102/956 (11)	1/956 (< 0.5)	0/956 (0)
PP Week 4	28/136 (21)	8/136 (6)	0/136 (0)	2/136 (1)	5/135 [4 (1, 8)]	5/121 [4 (1, 9)]	57/879 (6)	0/879 (0)	0/879 (0)
PP Week 12	57/229 (25)	24/229 (10)	4/229 (2)	1/229 (< 0.5)	19/232 [8 (5, 12)]	19/198 [10 (6, 15)]	112/886 (13)	0/886 (0)	1/886 (< 0.5)
PP Week 24	91/378 (24)	29/378 (8)	3/378 (1)	3/378 (1)	22/379 [6 (4, 9)]	25/322 [8 (5, 11)]	108/841 (13)	4/841 (< 0.5)	0/841 (0)
PP Week 36	117/539 (22)	26/539 (5)	9/539 (2)	2/539 (< 0.5)	31/544 [6 (4, 8)]	26/457 [6 (4, 8)]	96/812 (12)	5/812 (1)	0/812 (0)
PP Week 48	127/689 (18)	25/689 (4)	4/689 (1)	1/689 (< 0.5)	29/691 [4 (3, 6)]	32/573 [6 (4, 8)]	71/779 (9)	0/779 (0)	0/779 (0)

*CI* Clopper-Pearson confidence interval, *AP* Antepartum, *PP* Postpartum, *PHQ-9* Patient health questionnaire 9, *aPSQI* Adapted pittsburgh sleep quality Index, *BPNS* Brief peripheral neuropathy screening

^a^Depression symptoms were evaluated using the PHQ-9 questionnaire

^b^Cognitive complaint was defined as responding “Yes, Definitely” to any of three questions relating to neurocognitive impairment

^c^Poor sleep quality was defined as aPSQI score ≥ 4

^d^Peripheral neuropathy was evaluated using the BPNS tool

### Prevalence of neurotoxicity

#### Probable depression

The percentage of women reporting at least mild depression symptoms (PHQ ≥ 5) peaked at 13% (95% CI: 9%, 18%) during PP Week 12 (Table 2). Probable depression (PHQ ≥ 10) peaked at 2% during PP Weeks 12 (1%, 5%) and 36 (1%, 4%; Supplemental Fig. 2).

#### *Cognitive complaint* and *Poor sleep quality*

The percentage of women reporting cognitive complaint and poor sleep quality also peaked at PP Week 12 (8% (5%, 12%) for cognitive complaint and 10% (6%, 15%) for poor sleep quality), then decreased to 4% (3%, 6%) for cognitive complaint and 6% (4%, 8%) for poor sleep quality at PP Week 48.

#### Peripheral neuropathy

Across all weeks, most women with peripheral neuropathy experienced a mild event (Grade 1). Peripheral neuropathy (Grade ≥ 1) peaked at 13% during PP Week 24 (11%, 16%; Supplemental Fig. 3).

### Study arm differences in neurotoxicity

#### Probable depression

Women in the Deferred INH arm had 1.41 (0.16, 17.12) times the odds of probable depression compared with women in the Immediate INH arm at PP Week 12 (Table 3). However, more women in the Immediate INH arm (7/270) reported symptoms of depression compared with women in the Deferred INH arm (4/269) at PP Week 36.

**Table 3:** Study arm differences in neurotoxicity (probable depression, cognitive complaint, poor sleep quality, and peripheral neuropathy) by analysis visit week. Values are presented as N/Total N [% (95% CI)] or OR (95% CI)

Analysis Visit	Probable Depression^a^			Cognitive Complaint^b^			Poor Sleep Quality^c^			Peripheral Neuropathy^d^		
	**Immediate INH**	**Deferred INH**	**OR**^**e**^ **(95% CI)**	**Immediate INH**	**Deferred INH**	**OR**^**e**^ **(95% CI)**	**Immediate INH**	**Deferred INH**	**OR**^**e**^ **(95% CI)**	**Immediate INH**	**Deferred INH**	**OR**^**e**^ **(95% CI)**
AP Visit	0/38 [0 (0, < 0.5)]	0/40 [0 (0, < 0.5)]	-	2/38 [5 (1, 18)]	3/40 [8 (2, 20)]	1.67 (0.26, 10.88)	5/36 [14 (5, 29)]	6/37 [16 (6, 32)]	1.31 (0.35, 4.82)	47/477 [10 (7, 13)]	56/479 [12 (9, 15)]	1.23 (0.81, 1.86)
PP Week 4	0/64 [0 (0, 6)]	2/72 [3 (0, 10)]	-	3/64 [5 (1, 13)]	2/71 [3 (0, 10)]	0.50 (0.08, 3.21)	1/58 [2 (0, 9)]	4/63 [6 (2, 15)]	4.25 (0.46, 39.68)	27/439 [6 (4, 9)]	30/440 [7 (5, 10)]	1.13 (0.66, 1.94)
**PP Week 12**	**2/111** **[2 (0, 6)]**	**3/118** **[3 (1, 7)]**	**1.41** **(0.16, 17.12)**	**6/113** **[5 (2, 11)]**	**13/119** **[11 (6, 18)]**	**2.19** **(0.80, 5.97)**	**8/97** **[8 (4, 16)]**	**11/101** **[11 (6, 19)]**	**1.36** **(0.52, 3.54)**	**53/439** **[12 (9, 15)]**	**60/447** **[13 (10, 17)]**	**1.14** **(0.77, 1.71)**
PP Week 24	3/186 [2 (0, 5)]	3/192 [2 (0, 4)]	0.97 (0.13, 7.35)	11/186 [6 (3, 10)]	11/193 [6 (3, 10)]	0.96 (0.41, 2.28)	13/159 [8 (4, 14)]	12/163 [7 (4, 13)]	0.89 (0.39, 2.02)	56/415 [13 (10, 17)]	56/426 [13 (10, 17)]	0.97 (0.65, 1.45)
PP Week 36	7/270 [3 (1, 5)]	4/269 [1 (0, 4)]	0.56 (0.12, 2.25)	14/272 [5 (3, 8)]	17/272 [6 (4, 10)]	1.23 (0.59, 2.54)	13/227 [6 (3, 10)]	13/230 [6 (3, 9)]	1.01 (0.46, 2.24)	49/402 [12 (9, 16)]	52/410 [13 (10, 16)]	1.05 (0.69, 1.60)
PP Week 48	3/342 [1 (0, 3)]	2/347 [1 (0, 2)]	0.66^**6**^ (0.05, 5.76)	10/344 [3 (1, 5)]	19/347 [5 (3, 8)]	1.93 (0.89, 4.22)	11/279 [4 (2, 7)]	21/294 [7 (4, 11)]	1.88 (0.89, 3.98)	38/389 [10 (7, 13)]	33/390 [8 (6, 12)]	0.86 (0.52, 1.40)

Immediate INH Group. Model adjusted for gestational age stratum at baseline did not converge

*OR* Odds ratio, *CI* Confidence interval, *INH* Isoniazid, *AP* Antepartum, *PP* Postpartum, *PHQ-9* Patient health questionnaire 9, *aPSQI* Adapted pittsburgh sleep quality index, *BPNS* Brief peripheral neuropathy screening

^a^Probable depression was defined as PHQ-9 score ≥ 10

^b^Cognitive complaint was defined as responding “Yes, Definitely” to any of three questions relating to neurocognitive impairment

^c^Poor sleep quality was defined as aPSQI score ≥ 4

^d^Peripheral neuropathy was defined as BPNS Grade ≥ 1

^e^OR represents the ratio of the odds of neurotoxicity in the Deferred INH Group to the Immediate INH Group, adjusted for gestational age stratum at baseline

^f^OR represents the ratio of the odds of neurotoxicity in the Deferred INH Group to the

#### Cognitive complaint and poor sleep quality

At PP Week 12, women in the Deferred INH arm had 2.19 (0.80, 5.97) times the odds of cognitive complaint and 1.36 (0.52, 3.54) times the odds of poor sleep quality compared with women in the Immediate INH arm.

#### Peripheral neuropathy

For peripheral neuropathy, women in the Deferred INH arm had 1.14 (0.77, 1.71) times the odds compared with women in the Immediate INH arm at PP Week 12. More women in the Deferred INH arm reported peripheral neuropathy compared with women in the Immediate INH arm, except at PP Week 24 and PP Week 48.

Of note, all 95% CIs for odds ratios included the null value of 1. Similar patterns in neurotoxicity across study arms were observed when stratified by EFV exposure status (Supplemental Table 3).

### Factors associated with neurotoxicity

#### Probable depression

At PP Week 36, women using Cotrimoxazole had 9.45 (1.32, 413.68) times the odds of probable depression compared with women not using Cotrimoxazole (Table 4).

**Table 4:** Baseline risk factors of neurotoxicity (probable depression, cognitive complaint, poor sleep quality, and peripheral neuropathy). Values are presented as adjusted OR (95% CI)

Risk Factor	Adjusted^a^ OR (95% CI)			
	**Probable Depression**^**b,c**^	**Cognitive Complaint**^**b,d**^	**Poor Sleep Quality**^**b,e**^	**Peripheral Neuropathy**^**f,g**^
Entry Cotrimoxazole Use	9.45 (1.32, 413.68)	1.95 (0.89, 4.26)	-	0.24 (0.16, 0.35)
Positive Entry Hepatitis C serology	-	-	-	5.83 (1.32, 25.75)
CD4 Count < 350^h^	-	0.33 (0.09, 1.20)	-	-
350—< 500^h^	-	1.09 (0.47, 2.57)	-	-
500—< 650^h^	-	0.60 (0.20, 1.76)	-	-
HBsAg + at Entry	-	-	-	1.61 (0.70, 3.72)
CD4 Count (cells/mm^3^)^i^	-	-	-	0.97 (0.94, 1.01)
Age (years)	-	-	-	1.03 (1.00, 1.06)
BMI (kg/m^2^)	-	-	-	1.06 (1.03, 1.10)

*OR* Odds ratio, *CI* Confidence interval, *EFV* Efavirenz, *HBsAg* Hepatitis B surface antigen, *BMI* Body mass index

^a^Multivariate model includes gestational age stratum and all covariates with *p* < 0.15 in simple models that were adjusted for gestational age stratum. Simple models included study arm, EFV-regimen, Hepatitis B surface antigen (HBsAg) status, Hepatitis C serology status, Country, CD4 count, HIV viral load, age, Body Mass Index (BMI), INH acetylation status, EFV metabolism status and Cotrimoxazole use. “– “ indicates that the covariate was not included in multivariate model

^b^Outcome measure at postpartum Week 36

^c^Probable depression was defined as PHQ-9 score ≥ 10

^d^Cognitive complaint was defined as “Yes, Definitely” to any of three questions relating to neurocognitive impairment

^e^Poor sleep quality was defined as aPSQI score ≥ 4

^f^Peripheral neuropathy was defined as BPNS Grade ≥ 1

^g^Outcome measure at any study visit

^h^Reference group was CD4 Count ≥ 650

^i^Per 50 cells/mm^3^ increase

**Table 5:** Regulatory bodies that approved the P1078 study

Country	IRBs
South Africa	­ University of Witwatersrand Research Ethics Committee ­ South African Health Products Regulation
Botswana	­ Botswana Ministry of Health -Health Research and Development Committee ­ Harvard University IRB - Harvard T.H. Chan School of Public Health Office of Regulatory Affairs and Research Compliance ­ Botswana Drug Regulatory Unit
India	­ Ethics Committee- B J Medical College and Sassoon General Hospitals
Tanzania	­ National Institute of Medical Research (NIMR)
Thailand	­ The Human Experimentation Committee, Research Institute for Health Sciences, Chiang Mai University ­ Research Ethics Committee, Faculty of Medicine, Chiang Mai University
Zimbabwe	­ Medical Research council of Zimbabwe ­ Medicine Control Authority of Zimbabwe ­ Research Council of Zimbabwe ­ Joint Research Ethics Committee for the University of Zimbabwe College of Health Sciences and Parirenyatwa Group of Hospitals

#### Cognitive complaint and poor sleep quality

Neither CD4 count group nor Cotrimoxazole use were significant risk factors for cognitive complaint at PP Week 36 in the multivariate model. At PP Week 36, no risk factors had a *P* value < 0.15 in the simple models.

#### Peripheral neuropathy

For peripheral neuropathy at any visit, HBsAg, hepatitis C serology, CD4 count, CD4 count group, age, BMI and Cotrimoxazole use were included in the multivariate model with gestational age stratum. Hepatitis C serology, age, BMI and Cotrimoxazole use were significant risk factors (*P* < 0.05) for peripheral neuropathy during the study period. Women with a positive hepatitis C serology (*n* = 9) had 5.83 (1.32, 25.75) times the odds of peripheral neuropathy compared with women who had negative hepatitis C serology. For each unit increase in BMI, the odds of peripheral neuropathy increased by 6% (3%, 10%), and for each 10-year increase in age, the odds of peripheral neuropathy increased by 37% (2%, 84%). Women using Cotrimoxazole had 0.24 (0.16, 0.35) times the odds of peripheral neuropathy compared with women not using Cotrimoxazole.

## Discussion

To our knowledge, this is the first paper that provides the frequencies of symptoms of neurocognitive toxicity, including symptoms of depression, poor sleep quality, cognitive dysfunction and peripheral neuropathy, in women with HIV on ART and initiating IPT in pregnancy or 12 weeks postpartum. The study provides an opportunity to determine potential associations between timing of INH for TB preventive therapy and neurotoxicity in the era of EFV-ART. Effects of timing of IPT initiation (during pregnancy versus postpartum) on neurotoxicity and associations of socio-demographic and medical factors with neurotoxicity were evaluated, with particular interest in IPT/EFV co-exposure.

The percentage of symptoms of neurocognitive toxicity observed in this study of 12% for depression, 10% for sleep disturbances and 8% for cognitive dysfunction were as expected for perinatal women in the general population. For postpartum depression, it is well documented that approximately 10–15% of all childbearing women are affected [9], arising from dysregulation of the endocrine axes related to mood changes [9]. However, the proportion of women with depression in this study was lower than observed in other perinatal cohorts of women living with HIV [21–24]. In IMPAACT P1078, the unexpectedly low frequency of depressive symptoms in women on neurotoxic drugs (INH and EFV) could be attributed to the extra care and support provided within the well-resourced clinical trial setting. Alternatively, the difference could be explained by the different assessments conducted. For example, Ngocho et al. used the Edinburgh Postnatal Depression Scale which is a screening tool with specificity lower than the PHQ-9 used in our study. [22]

The prevalence of poor sleep quality generally increases with age [25]. It is already established that people living with HIV experience more poor sleep quality compared to those without HIV [25, 26], and about 73% of adults with HIV report poor sleep quality symptoms [25]. However, there are limited data on the frequency of poor sleep quality in postnatal women living with HIV in low resourced settings. In a study by Gelaye et al*.*in pregnant women without HIV, 17% of antepartum women were classified as having poor sleep quality [27]. The unexpected lower frequency of 10% poor sleep quality reported in our study for postpartum women living with HIV is likely because of the younger age of the women in this study and the extra care and support provided in the research setting.

Cognitive dysfunction is common in people living with HIV, with up to 36% affected in a study involving non-pregnant adults living with HIV [28]. However, the study did not include pregnant and postnatal women. There are limited data on the frequency of cognitive dysfunction in postpartum women living with HIV and our study provides preliminary data. Our study also confirms previous reports showing association between hepatitis C infection and cognitive dysfunction [29, 30], strengthening the rationale for universal screening of pregnant women with HIV for co-infection with hepatitis C.

Peripheral neuropathy is the most common side effect of INH. The low prevalence of peripheral neuropathy observed in this study could be attributable to the recommended supplementation with pyridoxine [1] and that women with at least Grade 1 peripheral neuropathy were excluded from the study. Hepatitis C serology, age and BMI are factors already known to be associated with peripheral neuropathy [31]. However, the protective effect of Cotrimoxazole was an unexpected finding in our study and requires further investigation.

In our study, there was no evidence of an association between timing of initiation of IPT during antepartum and postpartum, concomitant with EFV use, and increased frequencies of probable depression, cognitive dysfunction, sleep disturbances or peripheral neuropathy. Pharmacokinetic data from South African pregnant and postpartum women living with HIV found that INH reduced EFV clearance, especially among those with slow NAT2 acetylator status [32]. Their study did not provide further data on whether the INH-induced increased EFV plasma concentration was associated with increased frequency of neurotoxicity symptoms in a non-pregnant population [7]. This P1078 exploratory analysis showed no evidence of effect modification with IPT and concomitant EFV use. However, despite the suggestion from the data in our study that IPT and EFV can be taken by postpartum women without fear of increased risk of symptoms of neurotoxicity, further study is advised to formally assess the drug-drug interactions using a larger sample size.

Cotrimoxazole was the only risk factor associated with probable depression. This association with depression is already documented [33]. Further study is advised to formally assess association of Cotrimoxazole with probable depression in peripartum women.

### Limitations

Given that the neurocognitive questionnaire was implemented after study initiation, all women do not have evaluations at every time point. Consequently, we are limited to cross-sectional analyses and between arm comparisons at some time points were underpowered due to the small number of events. Although we evaluated the incidence in the subset of women with at least one evaluation at both an antepartum and a postpartum visit (*n* = 77), analyses with this subset were underpowered due to the small number of events. Of the 27 women with probable depression, only two reported probable depression at more than one visit. Secondly, perinatal-specific thresholds are not available for the neurotoxicity tools in all countries in the trial, potentially overestimating symptoms common in pregnancy. Given the small number of women not on EFV, we had limited ability to formally assess effect modification of EFV. Thirdly, because a complete case analysis approach was used, all analyses assumed data were missing completely at random. However, the overall conclusions did not change after mean imputation of incomplete surveys in a sensitivity analysis. Finally, risk factor analyses occurred at PP Week 36 and at this time point, women in the early arm had already completed IPT.

## Conclusion

In our study, there was no evidence of an association between timing of initiation of IPT during antepartum and postpartum, concomitant use of EFV and increased frequencies of probable depression, cognitive dysfunction, sleep disturbances or peripheral neuropathy. Symptoms of neurotoxicity peaked at 12 weeks but was not higher compared to historical controls of post-partum women without HIV infection. Further study is advised to formally assess associations of risk factors for neurotoxicity.

Verbal screening for symptoms of neurocognitive toxicity is not costly and readily integrated with maternity services. Identifying the small percentage of women with moderate to severe depression and significant sleep disturbance or cognitive impairment is important in preventing the catastrophic events of poor mother-infant bonding and the adverse child development associated with maternal neurocognitive toxicity.

## Supplementary Information

**Additional file 1:** **Figure 1.** Copy of IMPAACT P1078 neurotoxicity questionnaire with PHQ-9, neurocognitive impairment, and aPSQI assessments. **Figure 2.** Distribution of PHQ-9 scores. **Figure 3.** Distribution of BPNS scores. **Table 1.** Maternal characteristics at baseline for the peripheral neuropathy and neurocognitive analysis sets. **Table 2.** Number of participants with neuro-cognitive toxicity evaluations by randomization group, INH exposure, and gestational age stratum. **Table 3.** Summary of neurotoxicity (probable depression, cognitive complaint, poor sleep quality, and peripheral neuropathy) by study arm and EFV exposure. **Table 4.** Incidence of neurotoxicity (probable depression, cognitive complaint, poor sleep quality, and peripheral neuropathy) by study arm.

## Data Availability

The data cannot be made publicly available due to the ethical restrictions in the study’s informed consent documents and in the International Maternal Pediatric Adolescent AIDS Clinical Trials (IMPAACT) Network’s approved human subjects protection plan; public availability may compromise participant confidentiality. However, data are available to all interested researchers upon request to the IMPAACT Statistical and Data Management Center’s data access committee by email to gro.frtsf@atad.cads or ude.dravrah@atad.cads. This committee reviews and responds to requests for data, obtains necessary approvals from IMPAACT leadership and the National Institutes of Health (NIH), arranges for signature of a Data Use Agreement, and releases the requested data.

## Abbreviations

AP: Antepartum
aPSQI: Adapted Pittsburg Sleep Quality Index
BMI: Body-Mass Index
BPNS: Bilateral Peripheral Neuropathy Screen
CI: Confidence Interval
DAIDS: Division of AIDS
DTG: Dolutegravir
EFV: Efavirenz
EFV-ART: Efavirenz -based Anti-Retroviral Therapy
IMPAACT: International Maternal, Pediatric, Adolescent AIDS Clinical Trials Network
INH: Isoniazid
IPT: Isoniazid Preventive Therapy
PHQ-9: 9-Question Patient Health Questionnaire
PSQI: Pittsburg Sleep Quality Index
PP: Post-partum
TB: Tuberculosis
