# Supplementary material for: Prevalence of neurotoxicity symptoms among postpartum women on isoniazid preventive therapy and efavirenz-based treatment for HIV: an exploratory objective of the IMPAACT P1078 randomized trial
Source: BMC Pregnancy Childbirth. 2023 Jan 17;23:34. doi: 10.1186/s12884-022-05341-3 (PMC9847058; doi:10.1186/s12884-022-05341-3)
Supplement: Supplementary file 1 — Additional file 1: Figure 1. Copy of IMPAACT P1078 neurotoxicity questionnaire with PHQ-9, neurocognitive impairment, and aPSQI assessments. Figure 2. Distribution of PHQ-9 scores. Figure 3. Distribution of BPNS scores. Table 1. Maternal characteristics at baseline for the peripheral neuropathy and neurocognitive analysis sets. Table 2. Number of participants with neuro-cognitive toxicity evaluations by randomization group, INH exposure, and gestational age stratum. Table 3. Summary of neurotoxicity (probable depression, cognitive complaint, poor sleep quality, and peripheral neuropathy) by study arm and EFV exposure. Table 4. Incidence of neurotoxicity (probable depression, cognitive complaint, poor sleep quality, and peripheral neuropathy) by study arm. [file 12884_2022_5341_MOESM1_ESM.docx]

**Supplemental Figure 1.** Copy of IMPAACT P1078 neurotoxicity questionnaire with PHQ-9, neurocognitive impairment, and aPSQI assessments.

**Supplemental Figure 2.** Distribution of PHQ-9 scores. Dotted line represents cut-off for probable depression (PHQ-9 ≥ 10), diamond represents mean PHQ-9 score. INH = isoniazid, PHQ-9 = Patient Health Questionnaire 9, AP = antepartum, PP = postpartum.

**Supplemental Figure 3.** Distribution of BPNS scores. Dotted line represents cut-off for peripheral neuropathy (BPNS ≥ 1), diamond represents mean BPNS score. INH = isoniazid, BPNS = Brief peripheral neuropathy screening, AP = antepartum, PP = postpartum.
